# Post-traumatic stress symptoms in hemodialysis patients with MERS-CoV exposure

**DOI:** 10.1186/s13030-020-00181-z

**Published:** 2020-04-15

**Authors:** A Jin Cho, Hong-Seock Lee, Young-Ki Lee, Hee Jung Jeon, Hayne Cho Park, Da-Wun Jeong, Yang-Gyun Kim, Sang-Ho Lee, Chang-Hee Lee, Kyung Don Yoo, Ae Kyeong Wong

**Affiliations:** 1 Department of Internal Medicine, Kangnam Sacred Heart Hospital, Hallym University College of Medicine, 1, Singil-ro, Yeongdeungpo-gu, Seoul, 07441 South Korea; 2 Hallym University College of Medicine, Psychiatry, Seoul, Republic of Korea; 3 Department of Internal Medicine, Armed Forces Capital Hospital, Seongnam, South Korea; 4 Internal Medicine, Kyung Hee University at Kangdong, Seoul, Republic of Korea; 5 Gangeung Medical Center, Anesthesiology, Gangeung, Republic of Korea; 6 Internal Medicine, Ulsan University Hospital, Ulsan, Republic of Korea

**Keywords:** Post-traumatic stress symptom, Hemodialysis, Middle East respiratory syndrome

## Abstract

**Background:**

Post-traumatic stress symptoms can occur in patients with medical illness. During the Middle East Respiratory Syndrome (MERS) outbreak in South Korea in 2015, some dialysis patients in three centers who were incidentally exposed to patients or medical staff with confirmed MERS-CoV infection were isolated to interrupt the spread of the infection. We aimed to investigate post-traumatic stress symptoms and risk factors among these patients.

**Materials and methods:**

In total, 116 hemodialysis (HD) patients in contact with MERS-CoV-confirmed subjects were isolated using three strategies, namely, single room isolation, cohort isolation, and self-quarantine. We used the Impact of Event Scale-Revised-Korean (IES-R-K) to examine post-traumatic stress symptoms at 12 months after the isolation period.

**Results:**

Of the 116 HD patients, 27 were lost to follow-up. Of the 89 patients, 67 (75.3%) completed the questionnaires. Single room isolation was used on 40 (58.8%) of the patients, cohort isolation on 20 (29.4%), and self-imposed quarantine on 8 (11.8%). In total, 17.9% of participants (*n* = 12) reported post-traumatic stress symptoms exceeding the IES-R-K’s cutoff point (≧18). Prevalence rates of IES-R-K ≧18 did not differ significantly according to isolation method. However, isolation duration was linearly associated with the IES-R-K score (standardized β coefficient − 0.272, *P* = 0.026). Scores in *Avoidance, Emotional numbing and Dissociation* subscale were higher in patients with longer isolation period.

**Conclusion:**

MERS was a traumatic experience for quarantined HD patients. IES-R-K scores were not significantly different by isolation methods. However, short isolation was associated with post-traumatic stress symptoms.

## Introduction

During the outbreak of the Middle East Respiratory Syndrome coronavirus (MERS-CoV) in South Korea in 2015, 186 confirmed cases were reported, including one patient with maintenance hemodialysis (HD); 36 (19.4%) of the patients died. In the course of coping with MERS-CoV, 16,752 people were quarantined. Some dialysis patients in three HD units were incidentally exposed to patients or healthcare workers with confirmed MERS-CoV infection. To interrupt the spread of MERS-CoV, these individuals were isolated from the community during the outbreak.

A life-threatening physical illness can lead to various psychological symptoms after recovery. In 2003, severe acute respiratory syndrome (SARS) spread across 30 countries, and those who were infected experienced social stigma and reported mental health problems such as anxiety, depression, and post-traumatic stress disorder (PTSD) [1–4]. In Hong Kong, the mental health of 1394 SARS survivors was assessed; 47.8% of the sampled participants experienced PTSD symptoms after recovery, and 25.6% of those who had PTSD symptoms continued experiencing mental health issues up to 30 months after the outbreak [5].

Patients with HD have a high risk of accompanying psychiatric illnesses such as depression, anxiety, and stress symptoms [6–8], and previous exposure to trauma indicates a greater risk of stress symptoms from a subsequent trauma [9]. In the outbreak of MERS-CoV in South Korea, some HD patients were quarantined irrespective of their own will to prevent secondary MERS-CoV infection. Fortunately, no further patients were infected by MERS-CoV; however, the possibility of a life-threatening infection and quarantine can be more traumatic to patients with chronic kidney disease than to the general population. The experience of those placed under quarantine in terms of compliance, emotional response, and psychological impact remains under-researched in HD patients. This study examined post-traumatic stress symptoms and associated factors among HD patients who were exposed to MERS-CoV-infected patients and isolated for a certain period.

## Methods

### Participants

A total of 116 HD patients were isolated in Kyung Hee University at Kangdong, Gangeung Medical Center and Kangdong Sacred Heart Hospital during the outbreak of MERS in Korea. Among those, 89 patients were included in this study because 27 were lost to follow-up at 12 months after quarantine. One HD patient and a head nurse in the HD room at the first two hospitals were confirmed to be MERS-CoV-infected. As a result, 107 HD patients at these two hospitals were suspected to have been exposed to the confirmed cases. In the third hospital, 9 HD patients were exposed to MERS-CoV from a confirmed case outside HD units. All three HD units performed isolation practice to prevent the further spread of MERS-CoV among maintenance HD patients.

### Isolation practice

According to the “Middle East respiratory syndrome clinical practice guideline for HD facilities” by the Korean Society of Nephrology during the MERS-CoV outbreak in 2015, a patient in close contact with a MERS patient, without fever or respiratory symptoms, would be subjected to hospitalized quarantine for 14 days since last exposure [10]. Close contact refers to a receiver of dialysis who was in the same place, at the same time as a suspected or confirmed MERS patient in the symptomatic period [11]. Asymptomatic casual contacts should be subjected to cohort isolation for 14 days after exposure and closely monitored for any suspicious symptoms. Casual contact refers to those who received dialysis on the same day, in the same room as a patient with suspected or confirmed MERS-CoV infection, but at different times and on different beds during the symptomatic period, without having worn appropriate personal protective equipment. Hospitalized quarantine was defined as single room or cohort isolation. Patients isolated in a single room received dialysis in their own rooms installed with dialysis machines. “Cohort isolation” is a method of hospitalized quarantine during HD treatment, in a shared HD room. Self-quarantine applied to a patient who received dialysis on a different day from dialysis date of MERS-CoV confirmed case or to patients who were exposed to MERS-CoV from a confirmed case outside HD units and had no respiratory symptoms. The patient was monitored for the development of fever or respiratory symptoms during quarantine.

### Clinical data and measures

Demographic information including age, gender, HD duration, history of diabetes mellitus, and previous cardiovascular diseaselaboratory data including hemoglobin, albumin, and hsCRP were collected at the time of enrollment.

A survey was conducted at 12 months after the isolation period. Post-traumatic stress symptoms were assessed with the Impact of Event Scale-Revised-Korean (IES-R-K), which showed good reliability and validity for the assessment of PTSD symptom severity [12]. The scale consisted of the following four factors: *intrusion; avoidance; hyperarousal*; and *sleep disturbance, emotional numbing, and dissociation*. A five-point Likert scale was used with response options ranging from “0 = Never” to “4 = Very often.” A high total score represented high severity of the symptoms; so, a total score of 18 or more was used as a cut-off [12]. The alpha coefficient for this sample was 0.873.

### Statistics

The data are expressed as mean ± standard deviation. Comparisons of continuous variables were performed using t-tests and paired t-tests. Categorical variables, expressed as percentages, were analyzed using the chi-square test. Univariate linear regression analyses were performed to analyze contributing factors towards high IES-R-K score. All calculations were performed using SPSS 18.0 (SPSS Inc. Armonk, NY). *P* < 0.05 was considered significant.

## Results

**Fig. 1:**
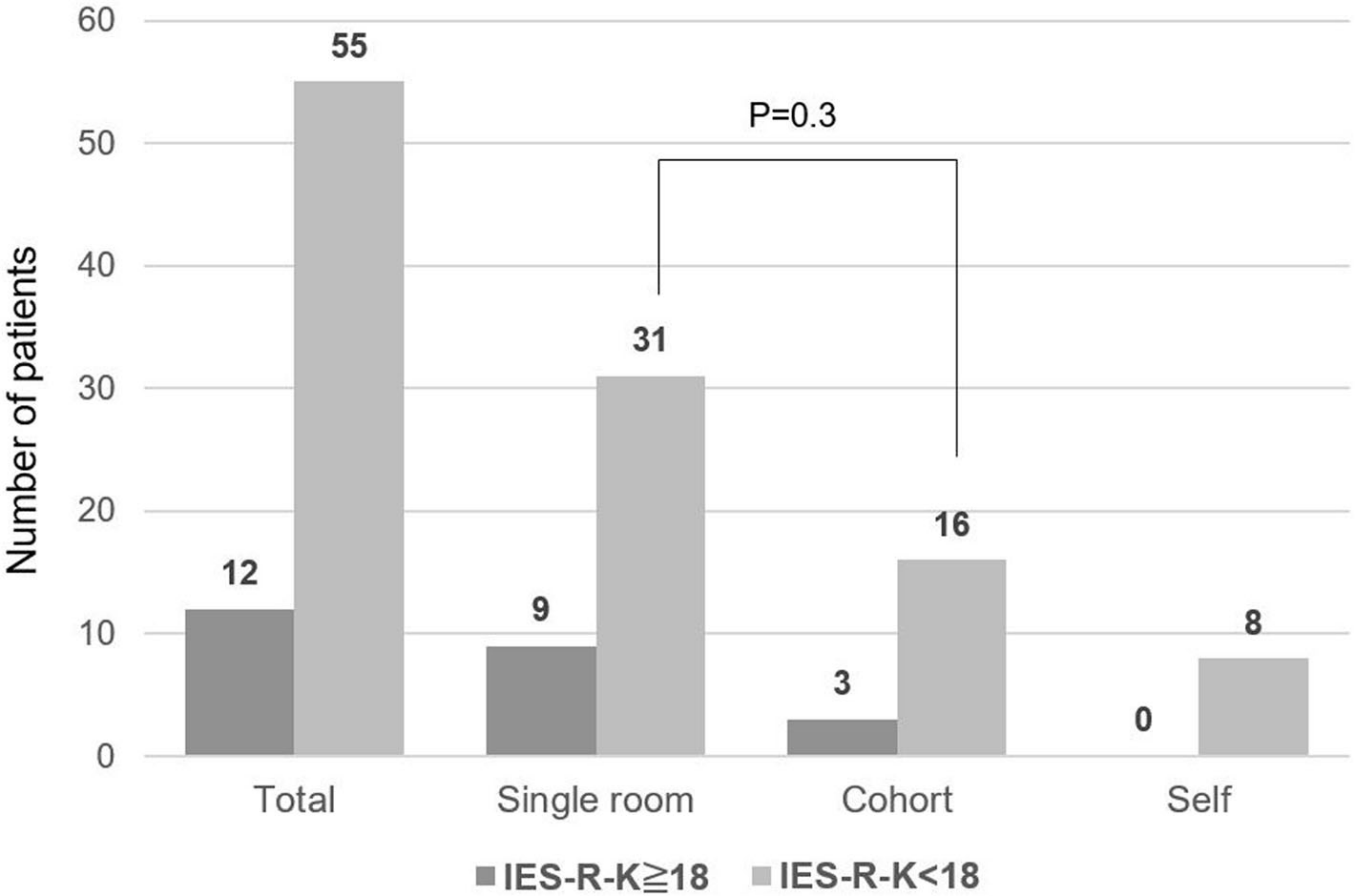
Prevalence of IES-R-K score≧18 in hemodialysis patients 1 year after MERS-CoV exposure

Of the 89 HD patients, 67 (75.3%) completed the questionnaires. Comparisons of respondents and non-respondents are shown in Supplementary Table 1. There was no significant difference between the two groups. The participants’ mean age was 62.6 years; 46 (68.7%) were men, 31 (46.3%) had diabetes, and 3 (4.4%) had a history of cardiovascular disease. The mean isolation period following exposure was 14.8 days. Single room isolation was implemented for 40 (58.8%) subjects; cohort isolation, for 20 (29.4%); and self-imposed quarantine, for 8 (11.8%) subjects. Figure 1 shows the prevalence of IES-R-K≧18 by isolation method. A total of 12 (17.9%) participants reported stress symptoms with IES-R-K scores exceeding cut-off point. Nine out of 40 patients (22.5%) in a single-room isolation group, 3 of 19 patients (15.8%) in a cohort-isolation group and none of the subjects in the self-imposed quarantine group were shown to have post-traumatic stress with IES-R-K≧18. The prevalence rate of IES-R-K≧18 did not differ significantly (*P* = 0.3) between the single-room and cohort-isolation groups. Table 1 shows a comparison of participants by IES-R-K cutoff point. Women and patients who had a shorter duration of isolation were more likely to develop symptoms of IES-R-K≧18.

**Table 1:** Baseline characteristics of participants according to IES-R-K score

Variables	IES-R-K≧18 *N* = 12	IES-R-K < 18 *N* = 55	*P*-value
Age (year)	59.4 ± 11.4	63.3 ± 13.3	0.4
Female, N (%)	7 (58.3)	14 (25.5)	0.03
Body mass index (kg/m^2^)	22.7 ± 4.2	23.0 ± 3.1	0.8
Hemodialysis duration (months)	47.2 ± 46.4	62.1 ± 62.6	0.4
Diabetes, N (%)	7 (58.3)	24 (43.6)	0.4
Previous cardiovascular disease, N (%)	1 (8.3)	2 (3.6)	0.5
Marital status^a^			
Married	6 (75)	31 (81.6)	0.7
Single or divorced	2 (25)	7 (18.4	
History of psychiatric consultation^a^	1 (12.5)	1 (2.6)	0.2
Family history of psychopathology^a^	0 (0)	1 (2.6)	0.6
Laboratory data			
Hemoglobin (g/dl)	10.2 ± 1.2	10.4 ± 1.0	0.7
Albumin (g/dl)	3.8 ± 0.5	3.7 ± 0.5	0.7
hsCRP (mg/dl)	4.0 ± 5.7	5.6 ± 8.0	0.7
Isolation			
Single room isolation, N (%)	9 (75)	31 (56.4)	0.3
Cohort isolation, N (%)	3 (25)	16 (29.1)	
Self-quarantine, N (%)	0 (0)	8 (14.5)	
Isolation duration (days)	13.0 ± 2.8	15.2 ± 3.0	0.02

Data expressed as mean (standard deviation) and number (percentage)

^a^46 of 67 subjects could be evaluated

We performed linear regression analysis to identify associated factors with high IES-R-K score (Table 2). Shorter isolation period was associated with high IES-R-K score (standardized β coefficient − 0.272, *P* = 0.026). We compared the total IES-R-K and subscale scores according to isolation duration in Table 3. The total scores did not differ significantly between male and female subjects (8.46 ± 9.59 vs. 12.38 ± 12.09, *P* = 0.158). However, *the sleep disturbance* subscale score was higher among female, compared to male subjects (1.57 ± 2.79 vs. 0.46 ± 1.17, *P* = 0.024). We divided the participants by median isolation duration. Participants isolated for less than 16 days showed a higher total score than those isolated for more than 16 days (13.47 ± 12.69 vs. 6.23 ± 6.45, *P* = 0.004). At a subscale level, *avoidance* (4.94 ± 5.94 vs. 2.34 ± 3.12, *P* = 0.027) and *sleep disturbance, emotional numbing, and dissociation* (4.75 ± 3.25 vs. 2.09 ± 2.28, *P* < 0.001) subscale scores were higher in participants who had been isolated for less than 16 days. With regard to the *sleep disturbance, emotional numbing, and dissociation* subscale scores, *emotional numbing* (2.22 ± 1.50 vs. 1.0 ± 1.16, *P* < 0.001) and *dissociation* (1.50 ± 1.59 vs. 0.49 ± 0.92, *P* = 0.002) scores were higher in that group.

**Table 2:** Linear regression analysis for IES-R-K score

Variables	Univariate	
	Standardized β Coefficient	*P*-value
Age (year)	−0.119	0.336
Female	0.175	0.158
DM	0.091	0.464
BMI (kg/m^2^)	−0.07	0.579
Dialysis duration (month)	−0.133	0.284
Isolation duration (day)	−0.272	0.026
Albumin (g/dl)	0.024	0.875

*Abbreviations*: *DM* Diabetes mellitus, *BMI* Body mass index

**Table 3:** IES-R-K score among study respondents (*N* = 67)

	Total		Sex		Isolation Duration	
			Male *N* = 46	Female *N* = 21	≧16 days *N* = 35	< 16 days *N* = 32
	Mean (SD)	95% CI	Mean (SD)	Mean (SD)	Mean (SD)	Mean (SD)
Total IES-R-K score	9.69 (1.28)	7.36–12.33	8.46 (9.59)	12.38 (12.09)	6.23 (6.45)^+^	13.47 (12.69)^+^
IES-R subscales						
Avoidance subscale	3.58 (0.59)	2.41–4.54	3.26 (4.76)	4.29 (5.01)	2.34 (3.12)^+^	4.94 (5.94)^+^
Intrusion subscale	1.78 (0.38)	0.97–2.59	1.54 (2.77)	2.29 (3.77)	1.31 (1.76)	2.28 (4.08)
Hyperarousal subscale	0.97 (0.27)	0.48–1.63	0.65 (1.98)	1.67 (2.63)	0.49 (1.22)	1.50 (2.91)
Sleep disturbance, emotional numbing, and dissociation subscale	3.36 (0.38)	2.62–4.18	3.00 (2.76)	4.14 (3.61)	2.09 (2.28)^++^	4.75 (3.25)^++^
- Sleep disturbance	0.81 (0.23)	0.36–1.23	0.46 (1.17)^+^	1.57 (2.79)^+^	0.60 (1.54)	1.03 (2.21)
- Emotional numbing	1.58 (0.18)	1.15–1.98	1.57 (1.51)	1.62 (1.36)	1.00 (1.16)^++^	2.22 (1.50)^++^
- Dissociation	0.97 (0.17)	0.68–1.29	0.98 (1.44)	0.95 (1.24)	0.49 (0.92)^+^	1.50 (1.59)^+^

^+^*P* < 0.05

^++^*P* < 0.001

Before the outbreak of MERS, 5.9% of the participants (*n* = 4) had scores exceeding the cutoff point of the IES-R-K. We did not have pre-MERS IES-R-K scores for 23 respondents. Therefore, a paired-samples t-test was conducted to compare pre- and post-MERS IES-R-K scores in only 44 respondents (Table 4). Participants’ post-traumatic stress symptoms increased at 1 year after the MERS outbreak. There was a significant difference in the total scores on the IES-R-K pre- and post-MERS (3.16 ± 9.02 vs. 9.69 ± 10.50, *P* < 0.001). At the subscale level, *sleep disturbance, emotional numbing, and dissociation* at post-MERS (2.52 ± 2.61) was significantly higher than that at pre-MERS (0.82 ± 2.40, *P* < 0.001). However, there was no significant difference in *avoidance*, *intrusion,* and *hyperarousal* at pre-MERS and post-MERS. When the fourth factor, which consists of three different symptoms, is broken down, there was a significant difference in *emotional numbing* (0.27 ± 0.85 vs. 1.16 ± 1.18, *P* < 0.001) and *dissociation* (0.20 ± 0.67 vs. 0.55 ± 0.93, *P* = 0.01) at pre- and post-MERS. However, *sleep disturbance* showed no significant difference (0.34 ± 1.36 vs. 0.82 ± 7.80, *P* = 0.07).

**Table 4:** IES-R-K score change before and after exposure to MERS-CoV (*N* = 44)

IES-R-K	N	Before	After	t	*P*-value
Hyperarousal	44	0.68 ± 2.39	0.91 ± 1.84	−0.57	0.6
Intrusion	44	0.98 ± 2.87	1.93 ± 2.97	−1.88	0.07
Avoidance	44	1.93 ± 4.69	3.32 ± 4.11	−1.90	0.06
Sleep disturbance, emotional numbing, and dissociation	44	0.82 ± 2.40	2.52 ± 2.61	−4.52	< 0.001
Sleep disturbance	44	0.34 ± 1.36	0.82 ± 1.80	−1.87	0.07
Emotional numbing	44	0.27 ± 0.85	1.16 ± 1.18	− 4.66	< 0.001
Dissociation	44	0.20 ± 0.67	0.55 ± 0.93	−2.71	0.01
Total	44	3.16 ± 9.02	9.69 ± 10.50	−4.73	< 0.001

Data expressed as mean (standard deviation)

Cases with missing values were excluded from the analysis

## Discussion

The present study examined post-traumatic stress symptoms from the quarantine experience of HD patients during the outbreak of MERS-CoV in Korea. In total, 17.9% of the patients developed stress symptoms with scores over the IES-R-K cut-off. Several studies have reported the psychological impact of the quarantine experience [13, 14]. Reynolds et al. showed that of the feelings experienced during SARS quarantine, boredom (62.2%), isolation (60.6%), and frustration (58.5%) were most commonly reported. In that study, an IES-R score of at least 20 was obtained by 14.6% of respondents [14]. In a study on the psychological effects of SARS quarantine in Toronto, the 129 quarantined persons who responded to a Web-based survey exhibited a high prevalence of psychological distress [13]. Symptoms of PTSD and depression were observed in 28.9 and 31.2% of respondents, respectively. In these studies, quarantine type was home or work. Subjects were not hospitalized.

The results of this study revealed that isolation duration is negatively associated with IES-R-K score. This result is contrary to those of previous studies that showed a positive correlation between quarantine duration and post-traumatic stress symptoms [13, 14]. However, isolation duration does not mean duration only, but also represents participants’ cooperativeness with the mandatory quarantine request. When HD patients who were exposed to MERS-CoV were asked to undergo mandatory quarantine, some were cooperative, such that they were admitted immediately following instructions by medical staff. Others resisted the isolation, dragging on until, finally, they were forcibly hospitalized at a later stage. Since all participants were later released from quarantine on the same day, the isolation duration indicated their cooperativeness with the mandatory quarantine and their trust in the prevention system of epidemics. Therefore, the results of this study indicated that participants who cooperated with the isolation request and received longer support reported fewer post-traumatic stress symptoms. At the subscale level, patients quarantined for less than 16 days showed higher *avoidance, emotional numbing, and dissociation*, compared to those quarantined for more than 16 days.

Psychological trauma causes not only post-traumatic stress symptoms such as *intrusion, avoidance, and hyperarousal*, but also many other somatic and psychiatric symptoms due to its complexity and the diversity of symptoms, showing high levels of comorbidity with other problems [15]. Based on Lee [16] suggestion regarding post-traumatic growth, trauma-related symptoms occur in sequential order, as follows: catatonia, emotional numbing, dissociation, fear, intrusion, paranoid ideation, avoidance, obsession, hyperarousal, anxiety, depression, existential emptiness, searching for meaning, and posttraumatic growth. The results of the current study, indicating that participants showed emotional numbing and dissociation more often, compared to intrusion, avoidance, and hyperarousal, in turn indicated that participants did not process the trauma and the isolation experience, so that their symptoms remained at the very early stage of processing trauma. This indicates that participants can experience subsequent symptoms of hyperarousal. Thus, psychological intervention and follow-up are needed.

In this study, being female was associated with post-traumatic stress symptoms. This is consistent with previous research that found gender differences in traumatized populations [17–20]. Specifically, the score on the *sleep disturbance* subscale was higher among female subjects. Before the outbreak of MERS, 5.9% of participants (*n* = 4) reported that they had pre-existing post-traumatic stress symptoms exceeding the cutoff point of the IES-R-K. This is higher than the current prevalence in the general population of a city in Korea (2.12%) [21]. This seems to be because the study sample comprised patients with HD, who are going through a life-threatening chronic disease.

There are several limitations in this study. First, this study had a low response rate and a small sample size. Therefore, the present findings may not be readily generalized to all isolated HD patients. Second, the IES-R-K is a self-report instrument tested 12 months after subjects’ exposure to MERS-CoV. Recall may have been affected and could have an impact on the reported results. Furthermore, other factors in addition to isolation experience might affect psychological stress in quarantined HD patients. Third, we had no information about subjects’ education level. Fourth, we did not measure other psychological and medical factors that could affect post-traumatic stress symptoms 12 months after isolation. Fifth, we didn’t investigate stress symptoms on HD patients without isolation as a control.

Despite these limitations, the results of this study show that quarantine can result in considerable psychological distress.. A shorter duration of quarantine was associated with high IES-R-K scores. Public health officials, infectious disease physicians, and psychologists must be aware of this result. Further research is needed to determine factors that influence the success of quarantine and in relation to the provision of additional support to patients who are at an increased risk of the adverse psychological effects of quarantine.

## Supplementary information

**Additional file 1: Table S1.** Comparison of study respondents with non-respondents.

## Data Availability

All data generated or analyzed during this study are included in this published article and its supplementary information files.
