# Supplementary material for: Post-traumatic stress symptoms in hemodialysis patients with MERS-CoV exposure
Source: Biopsychosoc Med. 2020 Apr 15;14:9. doi: 10.1186/s13030-020-00181-z (PMC7156895; doi:10.1186/s13030-020-00181-z)
Supplement: Supplementary file 1 — Additional file 1: Table S1. Comparison of study respondents with non-respondents. [file 13030_2020_181_MOESM1_ESM.docx]

Supplementary Table 1. Comparison of study respondents with non-respondents

| **Variables** | **Study respondents**  **N = 67** | **Non-respondents**  **N = 22** | **P-value** |
| --- | --- | --- | --- |
| Age (year) | 62.6 ± 13 | 56.8 ± 16.5 | 0.09 |
| Male, N (%) | 46 (68.7) | 11 (50.0) | 0.1 |
| Hemodialysis duration (months) | 59.4 ± 60.0 | 44.1 ± 32.5 | 0.3 |
| Diabetes, N (%) | 31 (46.3) | 10 (45.5) | 1.0 |
| Previous cardiovascular disease, N (%) | 3 (4.5) | 3 (13.6) | 0.1 |
| Laboratory data |  |  |  |
| Hemoglobin (g/dl) | 10.4 ± 1.0 | 10.1 ± 1.1 | 0.4 |
| Albumin (g/dl) | 3.7 ± 0.4 | 3.8 ± 0.5 | 0.7 |
| hsCRP (mg/dl) | 5.4 ± 7.6 | 6.6 ± 12.4 | 0.7 |
| Isolation |  |  |  |
| Single room isolation, N (%) | 40 (59.7) | 12 (54.4) | 0.1 |
| Cohort isolation, N (%) | 19 (28.4) | 10 (45.5) |  |
| Self-quarantine, N (%) | 8 (11.9) | 0 (0) |  |
| Isolation duration (days) | 14.8 ± 3.1 | 15.4 ± 3.0 | 0.4 |

Data were expressed as mean (standard deviation) and number (percentage).
